# Secretory autophagy and epithelial‐to‐mesenchymal transition in cadaveric AMD samples: Novel pathways in disease progression

**DOI:** 10.1111/aos.17558

**Published:** 2025-07-19

**Authors:** Iswariyaraja Sridevi Gurubaran, Ali Koskela, Hanna Heloterä, Kai Kaarniranta

**Affiliations:** ^1^ Department of Ophthalmology, Institute of Clinical Medicine University of Eastern Finland Kuopio Finland; ^2^ Department of Ophthalmology Kuopio University Hospital Kuopio Finland

**Keywords:** aging, drusen, epithelial degeneration, epithelial‐mesenchymal transition, macula, secretory autophagy

## Abstract

**Purpose:**

To examine the presence of secretory autophagy and epithelial–mesenchymal transition (EMT) in the macular retinal pigment epithelium (RPE) of human cadaver eyes with different forms of age‐related macular degeneration (AMD).

**Methods:**

Human cadaver macula samples representing dry and wet AMD, as well as age‐matched controls, were analyzed using immunohistochemistry. Markers of secretory autophagy, EMT, and inflammation were evaluated in RPE cells.

**Results:**

Increased expression of proteins associated with secretory autophagy and EMT was detected in the RPE of AMD samples compared to controls. These changes were observed in both dry and wet AMD forms.

**Conclusion:**

Secretory autophagy and EMT are elevated in the macular RPE of AMD‐affected eyes. These observations offer novel insight into AMD progression and potential therapeutic approaches.

To our knowledge, this is the first study to demonstrate that secretory autophagy and epithelial–mesenchymal transition (EMT) are simultaneously upregulated in different forms of age‐related macular degeneration (AMD) in human cadaver macula samples. Increased oxidative stress, mitochondrial and lysosomal dysfunction, protein aggregation and complement activation and inflammation are well‐established biomarkers of retinal pigment epithelial (RPE) cell degeneration (Kaarniranta et al., 2020). Phenotypic changes in the RPE cells and drusen accumulation are early clinical signs of AMD progression (Hyttinen et al., 2024; Kaarniranta et al., 2023).

Quiescent RPE cells are essential for regulating the visual cycle, including the clearance of photoreceptor outer segments in lysosomes and the degradation of damaged proteins and organelles. Lysosomal autophagy functions as a key cellular quality control mechanism, breaking down and recycling non‐functional proteins and damaged organelles (Kaarniranta et al., 2023). Autophagy‐related proteins regulate both conventional degradative autophagy and an unconventional secretory pathway. While degradative autophagy facilitates the breakdown of cellular components, secretory autophagy enables the controlled release of specific proteins, lipids and immune mediators into the extracellular space (Figure 1).

**FIGURE 1:**
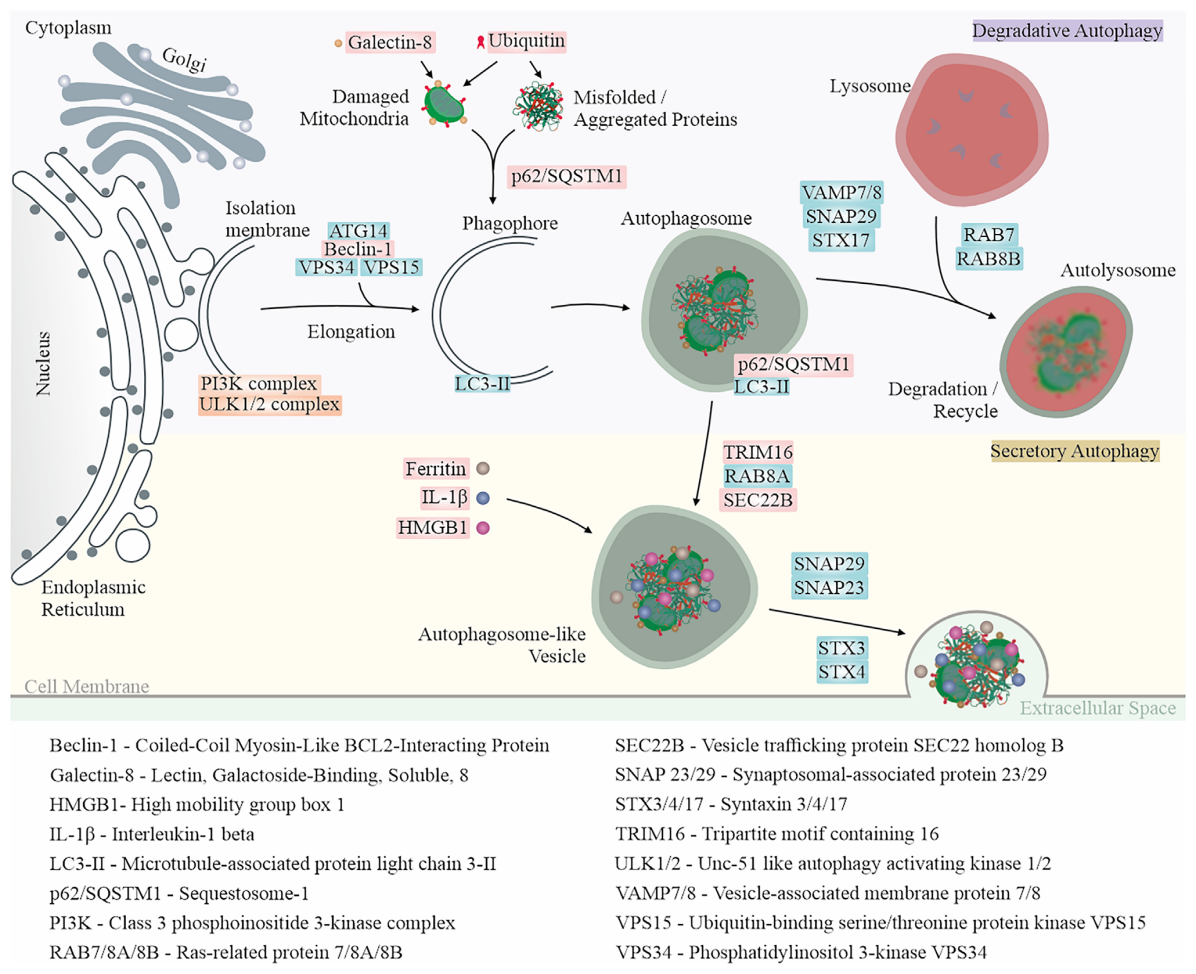
Autophagy is a self‐degradative process that can be either selective or non‐selective in removing cellular components. It is further classified into degradative autophagy and secretory autophagy. In degradative autophagy, damaged cellular components are ubiquitinated and marked by danger sensors such as galectin‐8 in the case of mitochondria and recognized by selective autophagy receptors, such as p62/SQSTM1, which facilitate cargo recognition. The formation of the isolation membrane (phagophore) involves the ULK1/2 complex, Beclin‐1, ATG14 and the PI3K complex (VPS15/VPS34). LC3‐II is then conjugated to the expanding phagophore membrane, enabling cargo sequestration. Once the autophagosome matures, it fuses with the lysosome, a process mediated by SNARE (soluble *N*‐ethylmaleimide‐sensitive factor attachment proteins receptor) proteins such as STX17, SNAP29, VAMP7/8 and the small GTPases RAB7 and RAB8B, leading to autolysosomal degradation of the enclosed material. In secretory autophagy, unique regulators such as TRIM16, SEC22B and RAB8A are involved in vesicle formation, trafficking and docking. SNARE proteins, including SNAP29/23 and STX3/4, mediate vesicle fusion with the plasma membrane. Unlike degradative autophagy, secretory autophagy mediates the secretion of leaderless (N‐terminal signal‐lacking) cytosolic proteins, complex particulate substrates and selectively exports specific cargos, such as Ferritin and particularly those involved in inflammation and immune responses, including IL‐1β and HMGB1.

EMT is a biological process in which polarized epithelial cells, such as the RPE cells, undergo biochemical changes that acquire a mesenchymal phenotype. This transition is characterized by increased extracellular matrix production, enhanced migratory capacity, invasiveness and resistance to apoptosis (Figure 2; Liukkonen et al., 2022; Kaarniranta et al., 2023).

**FIGURE 2:**
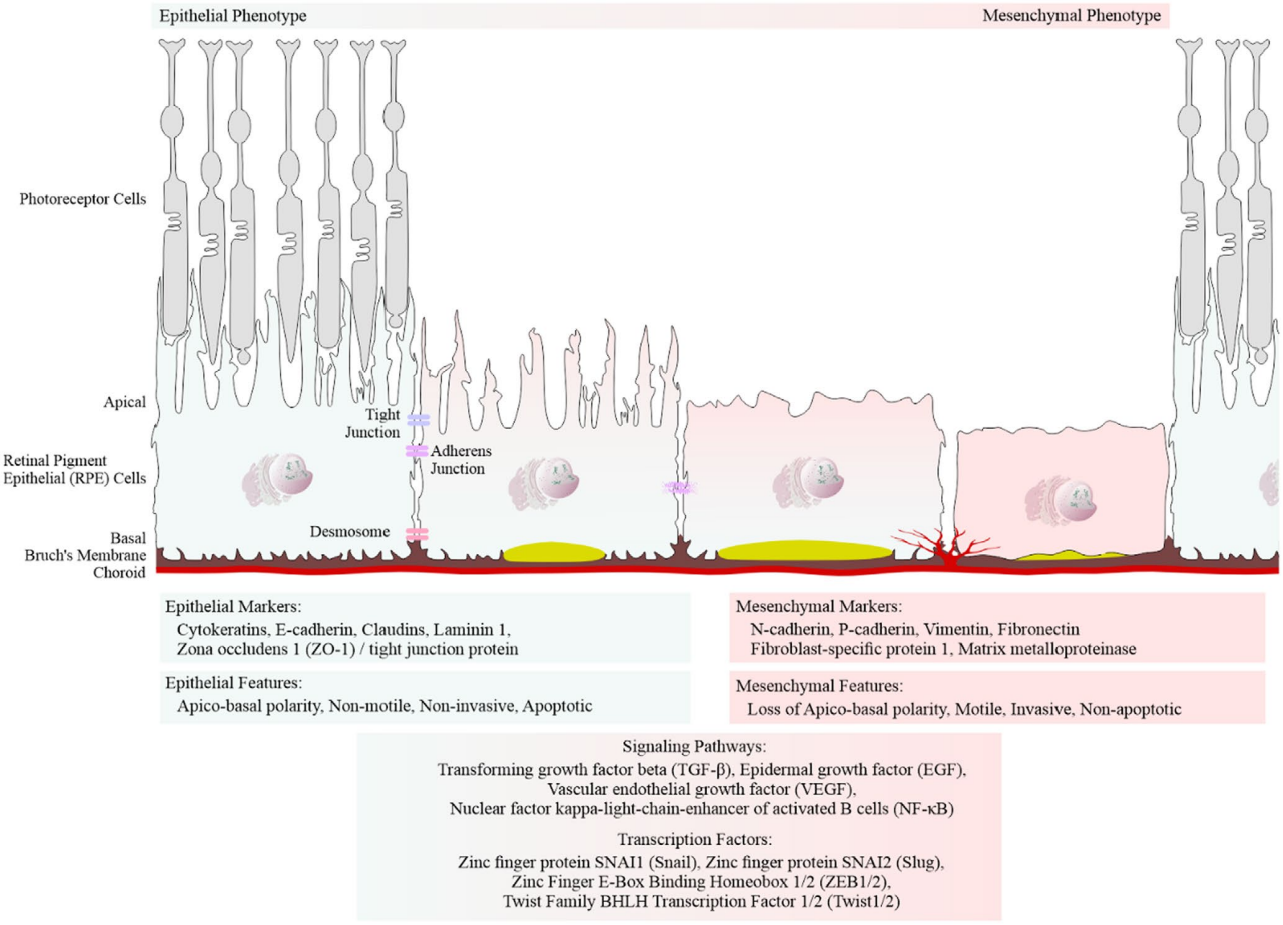
An epithelial–mesenchymal transition in the retinal pigment epithelial (RPE) cells. The RPE cells lose their polarity and cell–cell adhesion, acquiring mesenchymal traits in response to microenvironmental changes and pathological stimuli, particularly the disruption of interactions with Bruch's membrane.

Cadaveric eye tissues, collected postmortem, were used in this study. After enucleation, the eyes were fixed for 3 days at room temperature (RT) using a 10% formalin fixative from Reagena Oy (Catalogue No. 112256). Following primary fixation, the globes were horizontally dissected into three parts, post‐fixed in 10% formalin Avantor ScienceCentral™ (Catalogue No. VWRQ11699455) for 1.5 h at room temperature (RT) and then processed into paraffin. Parasagittal sections were cut from the central part of the globe, including the macular region. This included both the neurosensory retina and the underlying RPE. For immunohistochemical analysis, 5 μm parasagittal sections were used for staining. Detection of primary antibodies (Table 1) was performed using the Vector ImmPRESS® Polymer Kit, according to the manufacturer's protocol. Nuclei were counterstained with Haematoxylin QS (Catalogue No. H‐3404‐100‐NB). Brightfield images were acquired using Zeiss Axio Imager.M2 (Zeiss, DE), focusing on the RPE and neurosensory retina at 20× and 5× magnification, respectively. Imaging was performed using EC Plan‐Neofluar 20×/0.50 M27 and EC Plan‐Neofluar 5×/0.15 M27 lenses under identical microscope settings. Colour intensity was quantified using ImageJ v1.54f with the colour deconvolution method. Data are presented as mean ± SEM. Statistical significance was assessed using the nonparametric Kruskal–Wallis test, followed by Dunn's multiple comparisons test. A *p*‐value <0.05 was considered statistically significant; ‘ns’ denotes not significant.

**TABLE 1:** List of primary antibodies.

Primary antibodies against	Host	Working dilution	Supplier catalogue number
Amyloid‐β	Mouse	1:250	MABN10
Beclin‐1	Rabbit	1:200	NB500‐249
C3	Rabbit	1:50	NBP2‐66994
C5	Rabbit	1:50	ab217027
Ferritin	Rabbit	1:300	MA5‐32244
Fibronectin	Mouse	1:100	sc‐271 098
Galectin‐8	Rabbit	1:100	PA5‐50965
HMGB1	Mouse	1:100	ab11354
Iba1	Rabbit	1:300	019‐19741
IL‐1β	Goat	1:100	AF401
N‐cadherin	Mouse	1:100	sc‐59987
p62/SQSTM1	Mouse	1:250	sc‐28359
SEC22B	Rabbit	1:200	ab181076
SLUG	Mouse	1:100	sc‐166476
TGF‐β2	Mouse	1:50	MAB612
TRIM16	Rabbit	1:100	PA5‐110515
Ubiquitin	Rabbit	1:100	Z0458
VEGF	Rabbit	1:100	RB‐9031‐P
Vitronectin	Mouse	1:50	sc‐74484
ZO‐1	Rat	1:50	sc‐33725

The study was approved by the Ethics Committee of Kuopio University Hospital (approval numbers 06/2006 and 42/2014) in accordance with the principles of the Declaration of Helsinki. Patient data were collected and securely stored in compliance with the European Union Regulation 2016/679 on personal data protection.

The sample groups were selected and characterized based on data from the Kuopio University Hospital Database, which provides a comprehensive record of individuals' lifetime medical history. For the control group, the selection of the left or right eye was based on the quality of tissue sections (Table 2). Control samples were obtained from cadaveric eyes that had no clinical or histological evidence of AMD. These samples were confirmed to lack AMD features, such as drusen, by examination of counterstained sections. In contrast, the dry AMD and wet (neovascular) AMD groups were defined based on documented clinical diagnoses. Further confirmation was provided by optical coherence tomography (OCT) images acquired during treatment (see Tables 3 and 4, Figure 3).

**TABLE 2:** Demographic characteristics of control group.

Control				
Sample No	ID	Gender	Eye	Age (years)
1	Case 1	Female	Left	92
2	Case 2	Male	Left	75
3	Case 3	Male	Left	88
4	Case 4	Male	Left	92
5	Case 5	Female	Right	87
6	Case 6	Male	Left	93
7	Case 7	Female	Right	88

**TABLE 3:** Demographic characteristics of dry AMD group.

Dry AMD				
Sample No	ID	Gender	Eye	Age (years)
1	Case 8	Female	Right	88
2	Case 9	Female	Left	74
3	Case 10	Female	Right	91
4	Case 11	Male	Right	94
5	Case 12	Female	Right	92
6	Case 13	Female	Right	95

**TABLE 4:** Demographic and treatment characteristics of wet AMD group.

Wet AMD							
Sample No	ID	Gender	Eye	Age (years)	Age at first injection (years)	Injections per year (*y*^)	Total No of injections
1	Case 14	Female	Left	88	83	y1 (*B3*); y2 (*0*); y3 (*0*); y4 (*0*); y5 (*0*); y6 (*0*); y7 (*0*); y8 (*0*); y9 (*0*); y10 (*0*); y11 (*0*); y12 (*0*)	Bevacizumab (3)
2	Case 15	Female	Left	74	84	y1 (*B9*); y2 (*B3, A3*); y3 (*B3, A1*); y4 (*B4*); y5 (*0*); y6 (*0*); y7 (*0*); y8 (*0*); y9 (*0*); y10 (*0*); y11 (*0*); y12 (*0*)	Bevacizumab (19) Aflibercept (4)
3	Case 16	Female	Left	91	85	y1 (*B2*); Outside of Wet AMD treatment Criteria	Bevacizumab (2)
4	Case 17	Female	Left	94	72	y1 (*R9*); y2 (*B1, R5*); y3 (*B4*); y4 (*B5*); y5 (*B5*); y6 (*B5*); y7 (*B6*); y8 (*B6*); y9 (*B6*); y10 (*B6*); y11 (*B3, A2*); y12 (*A1*); Outside of Wet AMD treatment Criteria	Bevacizumab (56) Aflibercept (3) Ranibizumab (14)

*Note*: ^: Consecutive years, B*: Bevacizumab, A*: Aflibercept, R*: Ranibizumab, *: No of injections.

**FIGURE 3:**
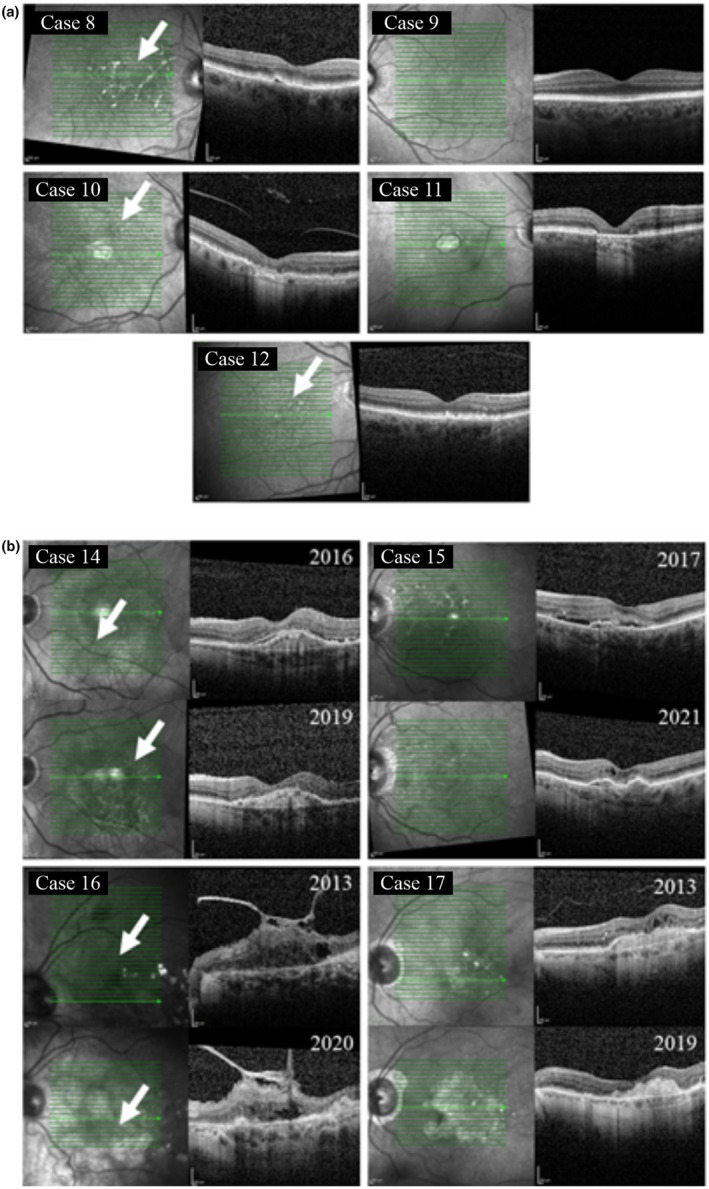
Optical coherence tomography (OCT) images from (a) dry AMD and (b) wet AMD cases, for which cadaver histological samples were collected. White arrows indicate drusen accumulations observed at various stages of AMD. The codes in the upper left panel correspond to the ID codes provided in Tables 3 and 4. In wet AMD cases, the year displayed in the upper right panel represents the date of the first OCT image acquired at Kuopio University Hospital Eye Clinic, while the year shown in the lower right panel represents the most recent OCT image available for each patient. Note the presence of geographic atrophy lesions in Case 10 and Case 11 and the absence of OCT data for Case 13. Images were acquired using the SPECTRALIS OCT system (Heidelberg Engineering, Heidelberg, Germany).

Our data revealed a significant increase in ubiquitin levels in the RPE cells from dry AMD, and an elevation in galectin‐8 levels in the RPE cells from both dry and wet AMD (Figures 1 and 4a,b). Furthermore, the ubiquitin‐binding protein p62/SQSTM1 was markedly upregulated in wet AMD compared to both the control group and dry AMD (Figure 5), while Beclin‐1 levels were significantly higher in the RPE cells from both dry and wet AMD relative to controls (Figure 6). These findings indicate an accumulation of damaged or non‐functional proteins and organelles that are targeted for degradation. The elevated expression of p62/SQSTM1 and Beclin‐1 suggests an enhanced activation of the degradative autophagy pathway. However, the concurrent accumulation of these damaged components may also be indicative of an insufficient degradative autophagic flux.

**FIGURE 4:**
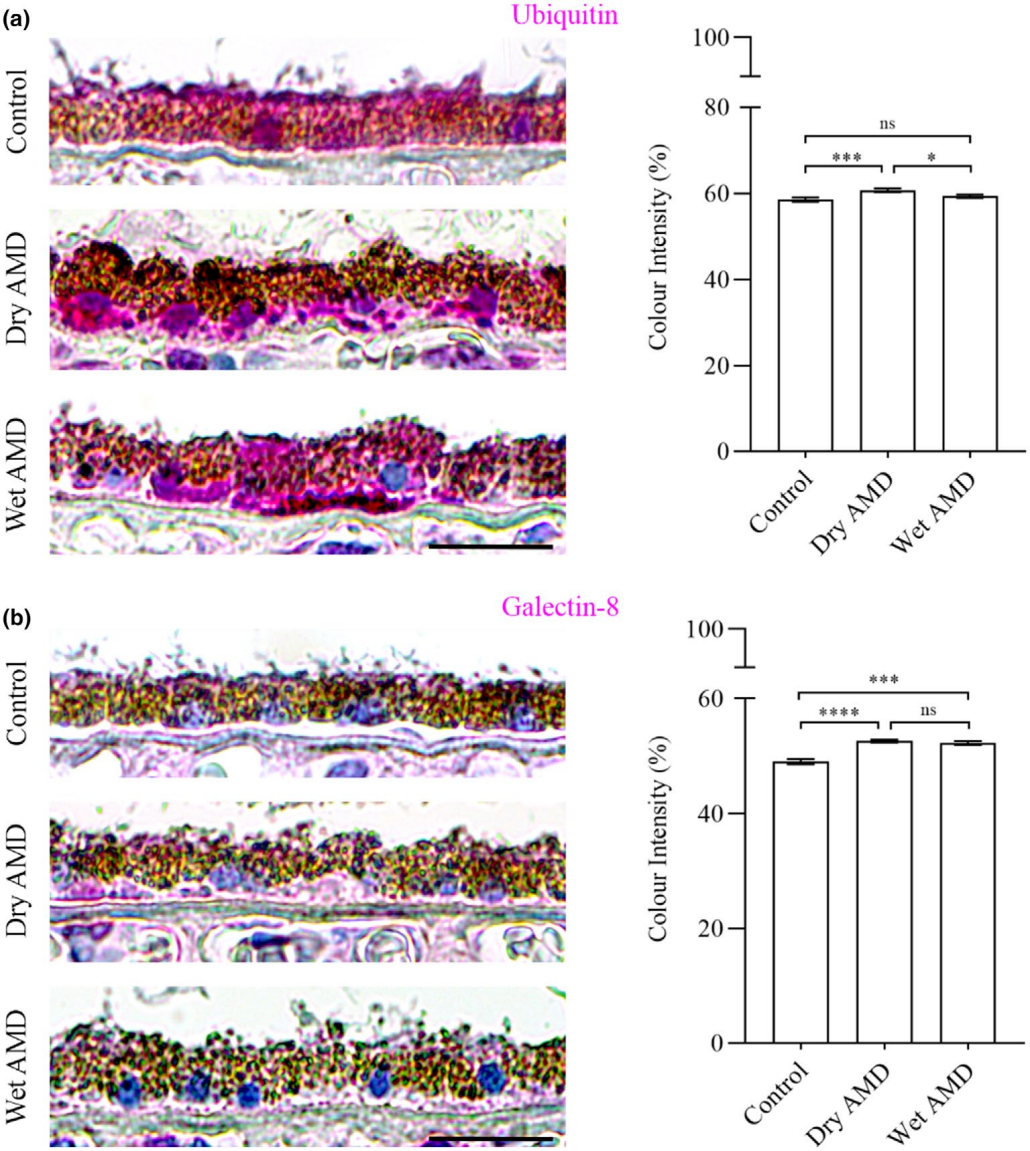
Immunohistochemical analysis of ubiquitin and galectin‐8 in the RPE cells from control, dry AMD and wet AMD tissue samples. (a) Ubiquitin levels showed a significant increase of ~4% in dry AMD, while no significant change was observed in wet AMD (~1.5% increase) compared to the control group. Additionally, ubiquitin levels were ~2% higher in dry AMD compared to wet AMD. (b) Galectin‐8 levels were significantly elevated in dry AMD (~7%) and wet AMD (~6.5%) compared to controls. However, no significant difference was observed between dry and wet AMD, with wet AMD showing a slight (~0.5%) decrease in galectin‐8 levels relative to dry AMD. Scale bar = 5 μm. ****p = 0.001, ***p = 0.009 & 0.003, *p = 0.041, ns, not significant.

**FIGURE 5:**
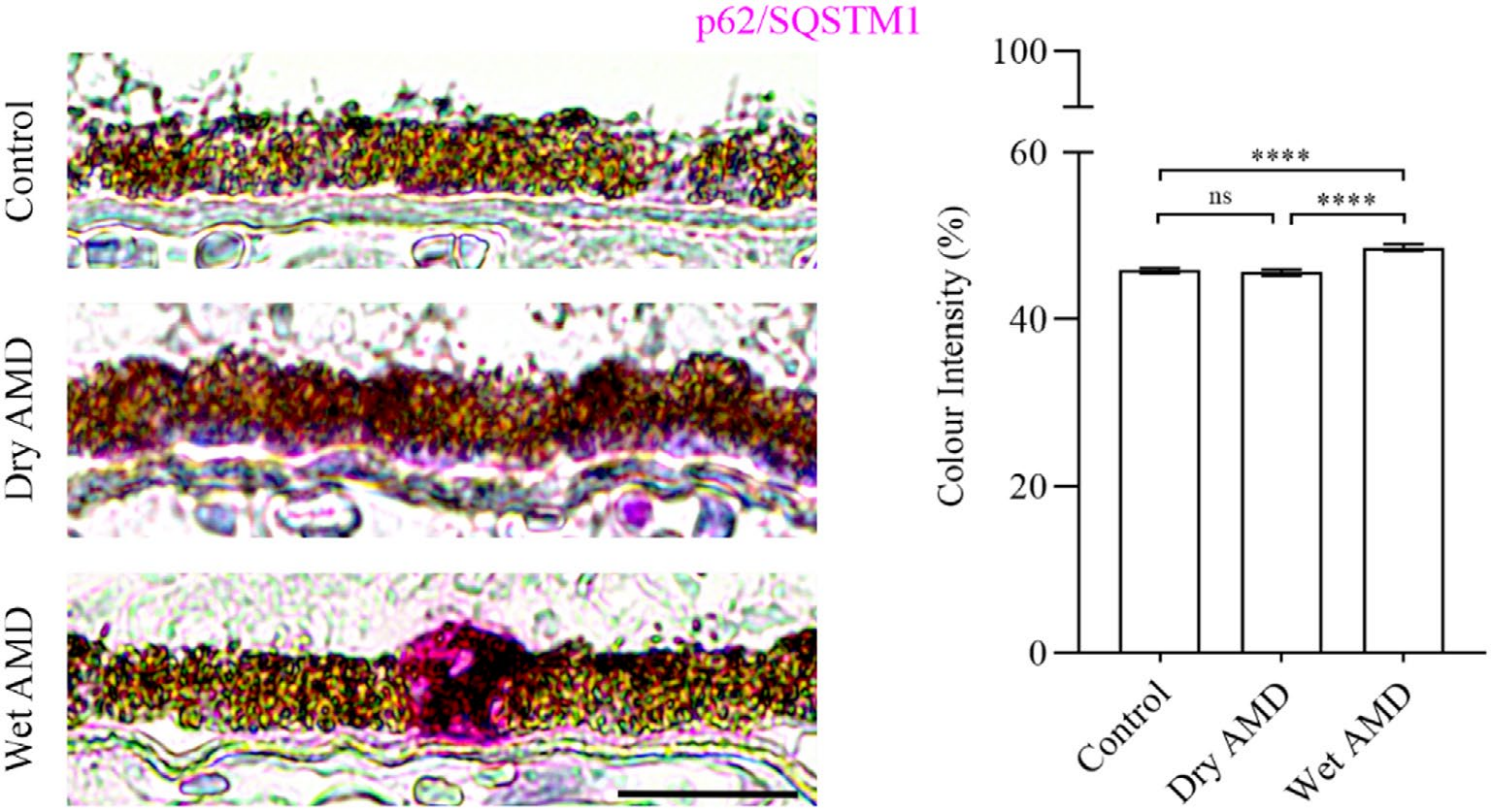
Immunohistochemical analysis of p62/SQSTM1 in the RPE cells from control, dry AMD and wet AMD tissue samples. Compared to controls, p62/SQSTM1 expression showed a slight but non‐significant decrease (~1.5%) in dry AMD. In contrast, wet AMD samples exhibited a significant increase of ~12% relative to controls and ~13% compared to dry AMD. Scale bar = 5 μm. ****p = 0.001, ns, not significant.

**FIGURE 6:**
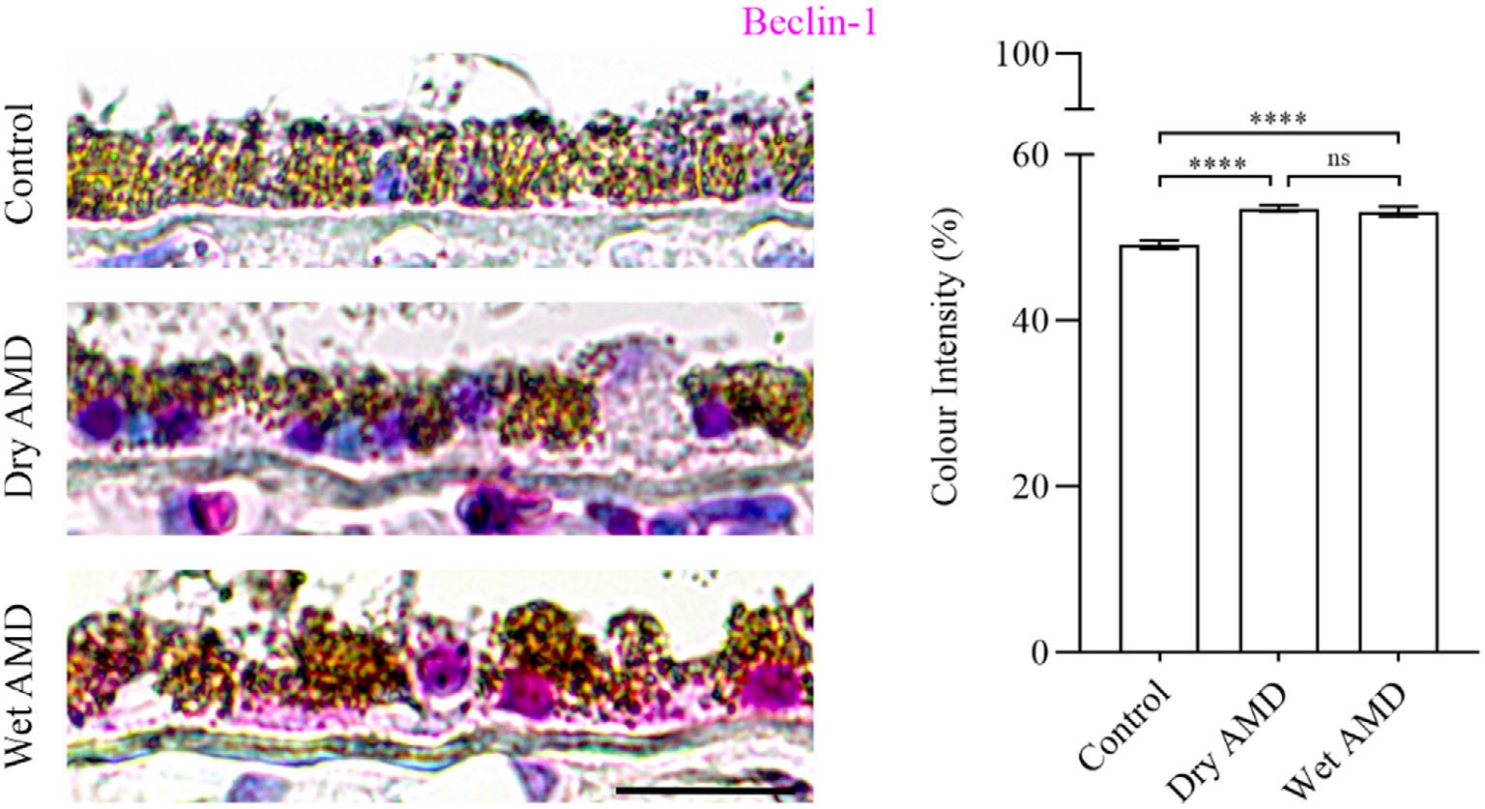
Immunohistochemical analysis of Beclin‐1 in the RPE cells from control, dry AMD and wet AMD tissue samples. Beclin‐1 levels were significantly elevated in dry AMD (~8.9%) and wet AMD (~8%) compared to the control group. However, there was no significant difference between dry and wet AMD, with wet AMD showing a slight (~0.7%) decrease relative to dry AMD. Scale bar = 5 μm. ****p = 0.001, ns, not significant.

Next, we analysed TRIM16 and SEC22B, which are specifically involved in secretory autophagy, including cargo recognition and fusion with the plasma membrane. TRIM16 levels were significantly increased in both dry and wet AMD compared to the control group (Figure 7a). Similarly, SEC22B expression was markedly elevated in both dry and wet AMD relative to controls (Figure 7b), suggesting a strong upregulation of secretory autophagy in both dry and wet AMD.

**FIGURE 7:**
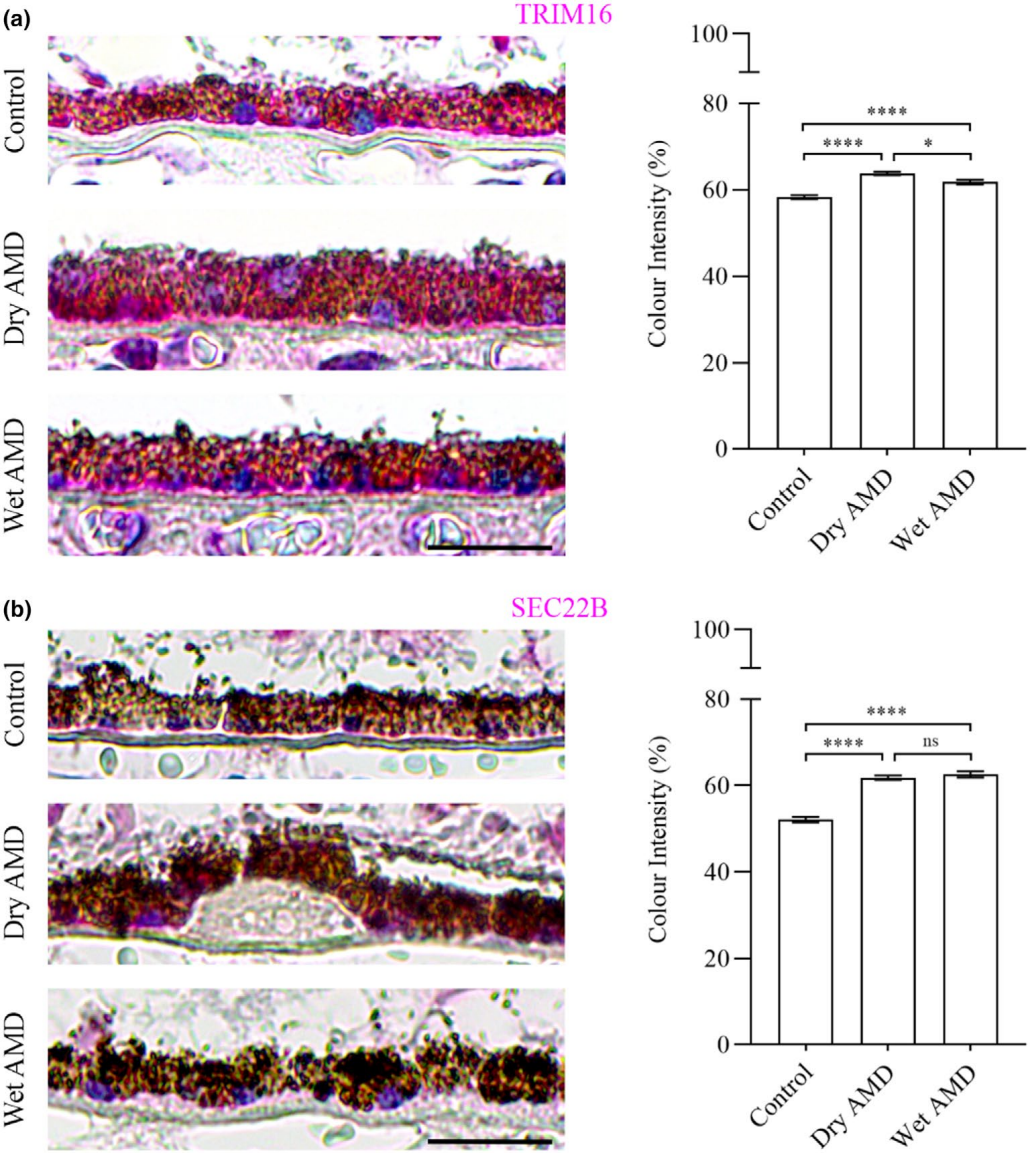
Immunohistochemical analysis of TRIM16 and SEC22B in the RPE cells from control, dry AMD and wet AMD tissue samples. (a) TRIM16 expression was significantly increased by ~9.5% in dry AMD and ~6% in wet AMD compared to controls. Additionally, TRIM16 levels were ~3% higher in dry AMD than in wet AMD. (b) SEC22B expression was significantly elevated in dry AMD (~18.5%) and wet AMD (~20%) compared to controls. However, no significant difference was observed between dry and wet AMD, with SEC22B levels in wet AMD showing a slight (~1%) increase relative to dry AMD. Scale bar = 5 μm. ****p = 0.001, *p = 0.018, ns, not significant.

Secretory autophagy plays a role in the secretion of specific cargos, including Ferritin, which regulates iron homeostasis, the proinflammatory cytokine IL‐1β and the nuclear protein HMGB1, which acts as a danger signal contributing to inflammation and cell migration. In our study, we observed a marked increase in Ferritin (Figure 8a), IL‐1β and HMGB1 levels in both dry and wet AMD compared to controls (Figure 8b,c). These findings align with the elevated activity of secretory autophagy in AMD, further supporting its role in disease progression.

**FIGURE 8:**
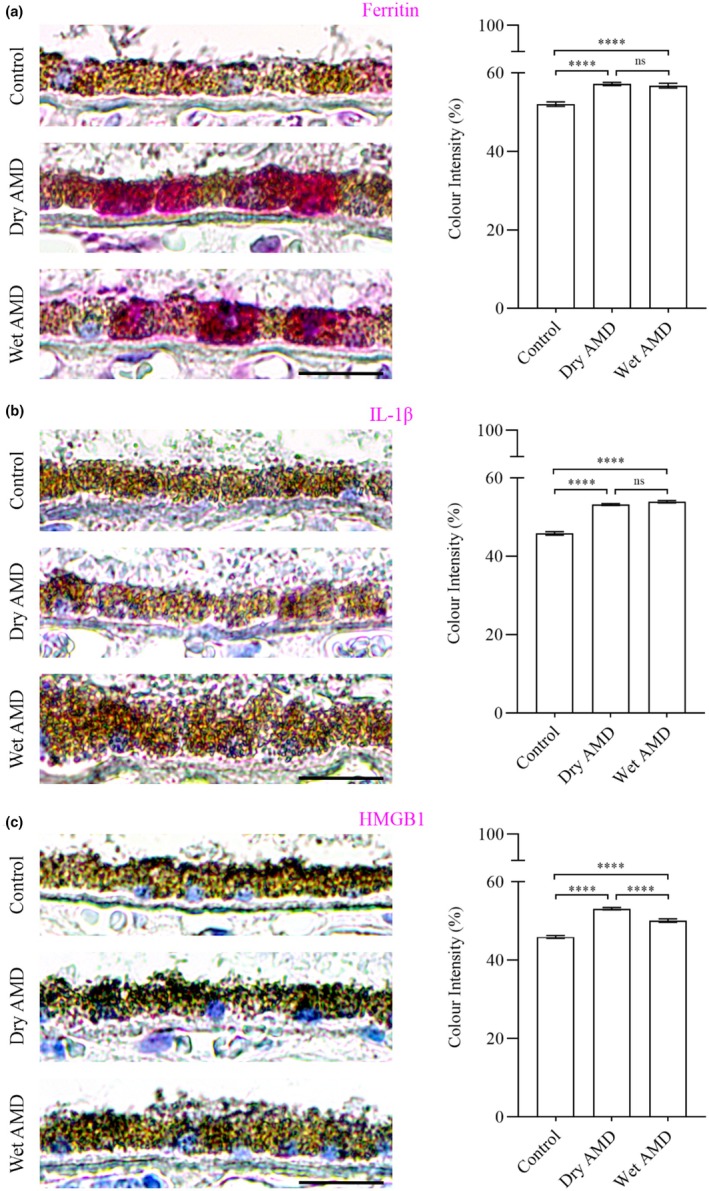
Immunohistochemical analysis of Ferritin, IL‐1β and SEC22B in the RPE cells from control, dry AMD and wet AMD tissue samples. (a) Ferritin levels were significantly increased in dry (~10%) and wet (~9%) AMD compared to controls, with a slight (~0.7%) decrease in wet AMD relative to dry AMD. (b) IL‐1β levels were significantly elevated in dry (~16%) and wet (~17.6%) AMD compared to controls. Wet AMD exhibited a non‐significant (~1.3%) increase compared to dry AMD. (c) HMGB1 levels were significantly increased in dry (~16%) and wet (~9%) AMD relative to controls. However, wet AMD showed a significant (~6%) decrease in HMGB1 compared to dry AMD. Scale bar = 5 μm. ****p = 0.001, ns, not significant.

Additionally, we analysed the major drusen‐associated aggregation protein, amyloid‐β and the extracellular matrix component, Vitronectin, in dry and wet AMD compared to control groups (Figure 9a,b).

**FIGURE 9:**
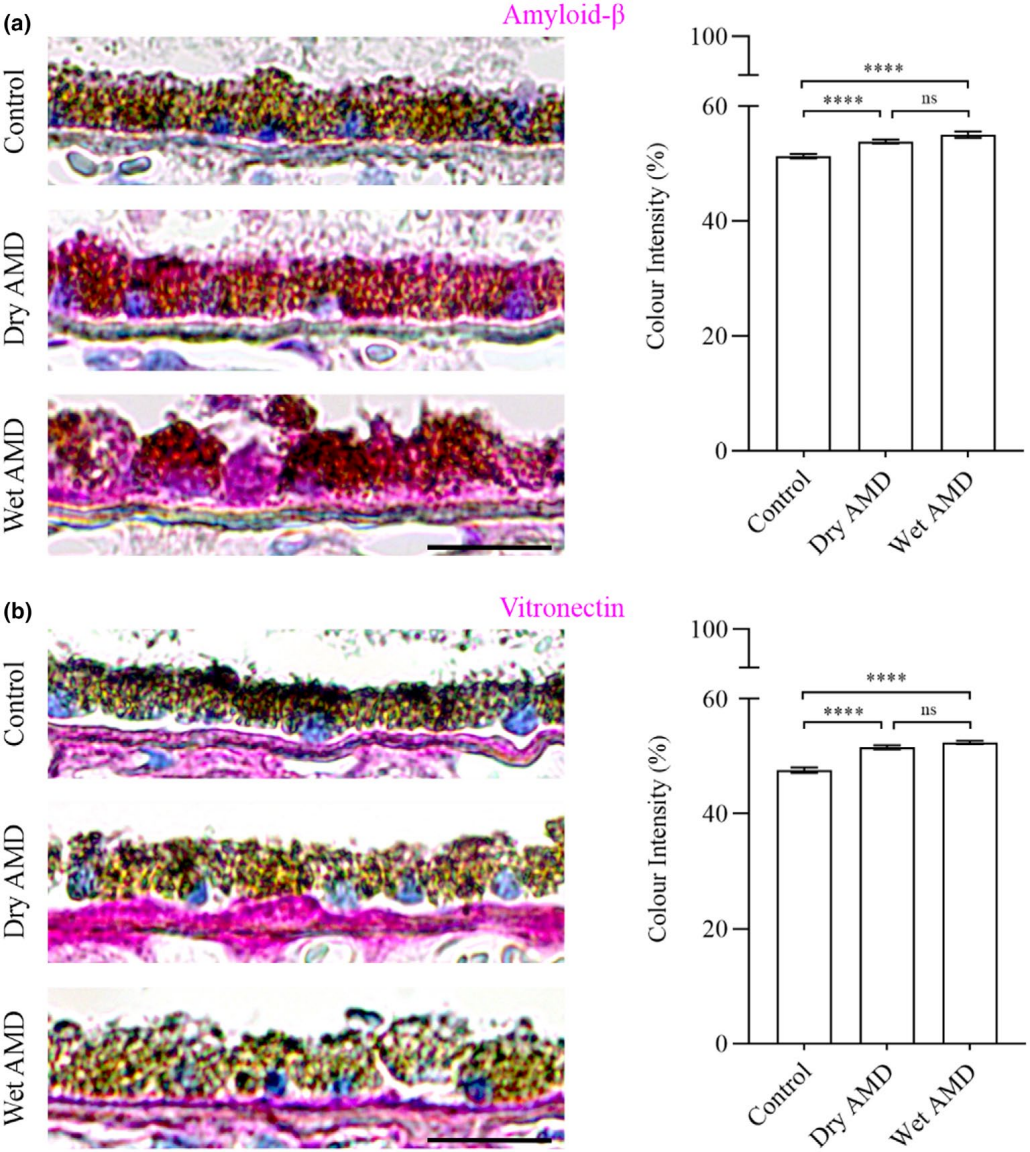
Immunohistochemical analysis of amyloid‐β and Vitronectin in the RPE cells from control, dry AMD and wet AMD tissue samples. (a) Amyloid‐β expression was significantly increased by ~5% in dry AMD and ~7% in wet AMD compared to controls. Additionally, amyloid‐β levels were ~2% higher in wet AMD than in dry AMD, although the difference was not statistically significant. (b) Vitronectin expression was significantly elevated in dry AMD (~8%) and wet AMD (~10%) compared to controls. However, no significant difference was observed between dry and wet AMD, with Vitronectin levels in wet AMD showing a slight (~1.6%) increase relative to dry AMD. Scale bar = 5 μm. ****p = 0.001, ns, not significant.

Furthermore, we performed immunostaining for the tight junction protein ZO‐1 (Figure 10a) and the mesenchymal markers N‐cadherin and fibronectin in control, dry AMD and wet AMD tissue samples (Figures 2 and 10b,c). Our analysis revealed a significant increase in ZO‐1, N‐cadherin and fibronectin levels in both dry and wet AMD compared to the control group (Figure 10).

**FIGURE 10:**
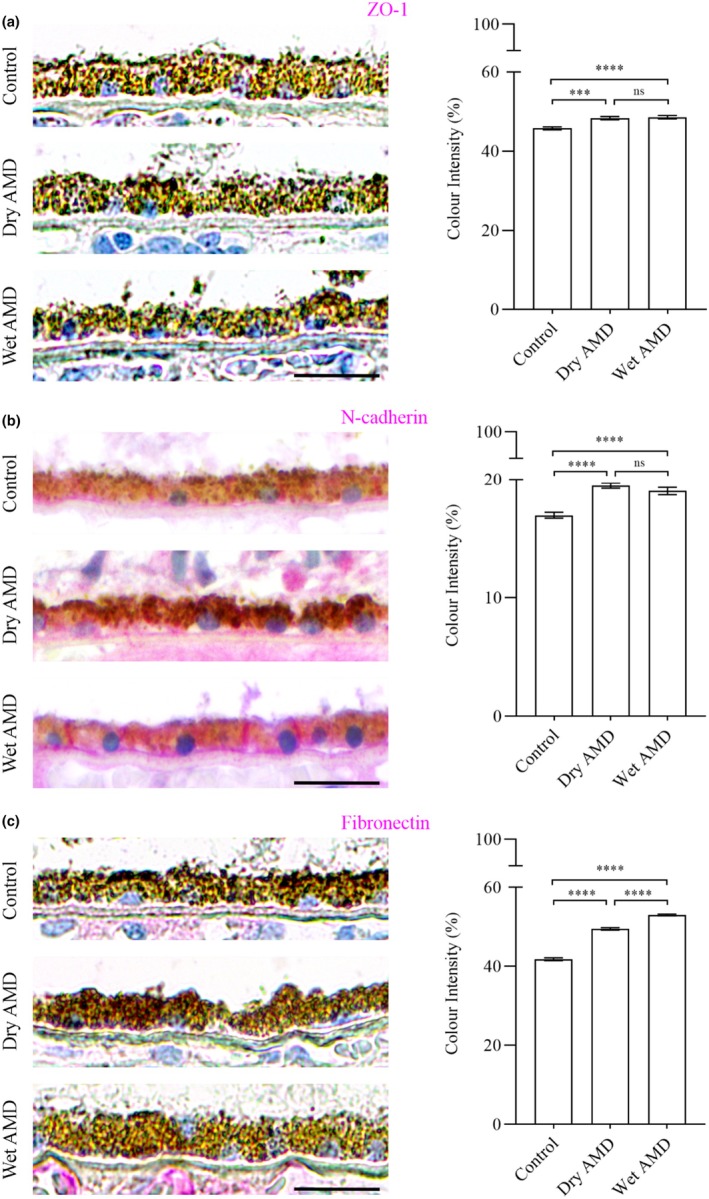
Immunohistochemical analysis of tight junction protein ZO‐1 and mesenchymal markers N‐cadherin and fibronectin in the RPE cells from control, dry AMD and wet AMD tissue samples. (a) ZO‐1 expression was significantly increased in dry AMD (~5%) and wet AMD (~6%) compared to controls. Additionally, wet AMD showed a non‐significant ~0.5% increase relative to dry AMD. (b) N‐cadherin levels were significantly elevated in dry AMD (~14.7%) and wet AMD (~12%) compared to controls. Wet AMD exhibited a non‐significant ~2.3% decrease compared to dry AMD. (c) Fibronectin expression was significantly increased in dry AMD (~18%) and wet AMD (~26%) compared to controls, with a significant ~7% increase in wet AMD relative to dry AMD. Scale bar = 5 μm. ****p = 0.001, ***p = 0.002, ns, not significant.

The analysis of EMT regulators, including TGFβ‐2 (Figure 11a), VEGF (Figure 11b) and the transcription factor SLUG (Figure 11c), revealed a significant increase in both dry and wet AMD compared to the control group. These findings align with the upregulation of circulatory EMT markers, indicating a potential role of EMT in AMD pathophysiology (Liukkonen et al., 2022).

**FIGURE 11:**
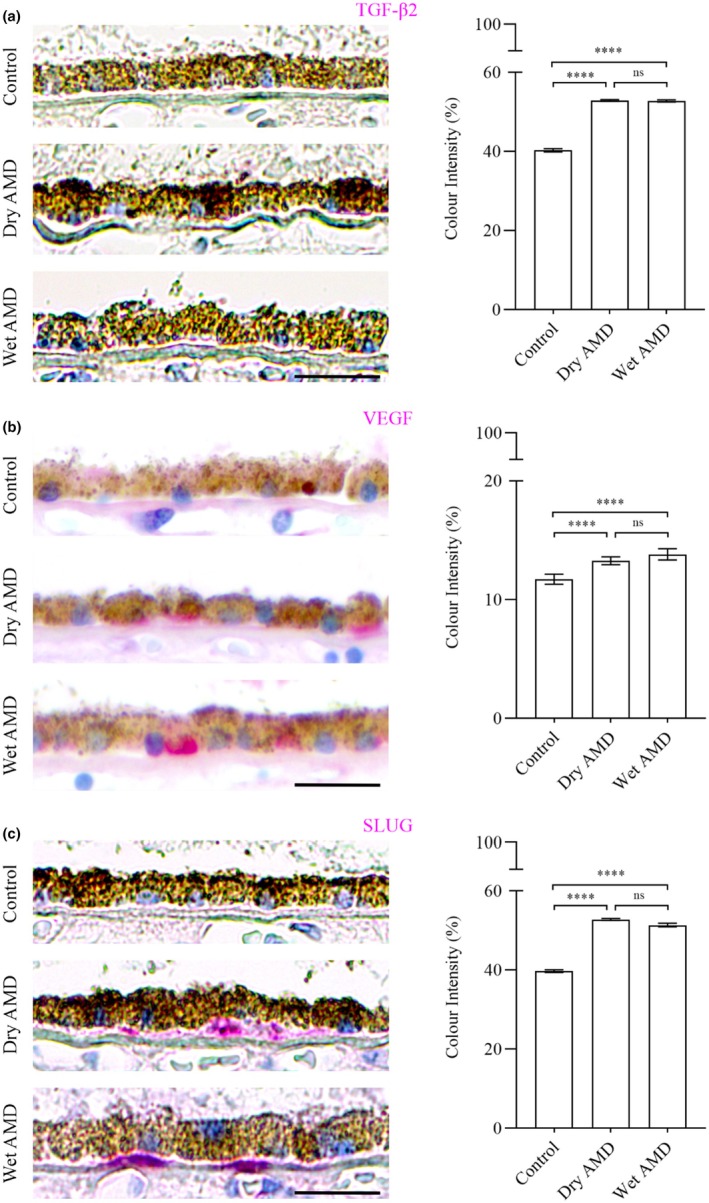
Immunohistochemical analysis of EMT regulators TGFβ‐2, VEGF and SLUG in the RPE cells from control, dry AMD and wet AMD tissue samples. (a) TGFβ‐2 expression was significantly elevated in dry (~31%) and wet (~30.9%) AMD compared to controls, with a slight (~0.2%) decrease in wet AMD relative to dry AMD. (b) VEGF levels were significantly increased in dry (~13%) and wet (~17%) AMD compared to controls, with a non‐significant ~4% increase in wet AMD compared to dry AMD. (c) SLUG expression was significantly upregulated in dry (~32%) and wet (~29%) AMD compared to controls, showing a non‐significant ~2.6% decrease in wet AMD relative to dry AMD. Scale bar = 5 μm. ****p = 0.001, ns, not significant.

Lastly, we analysed inflammatory markers, including complement components C3, C5 (Figure 12) and the microglial marker Iba1 (Figure 13). We observed no differences in C3 levels between control, dry and wet AMD (Figure 12a). In contrast, C5 levels were significantly increased in both dry and wet AMD compared to the control group (Figure 12b). We observed sporadic Iba1 positivity in the control retina with consistent positivity in both dry and wet retinal tissues (Figure 13).

**FIGURE 12:**
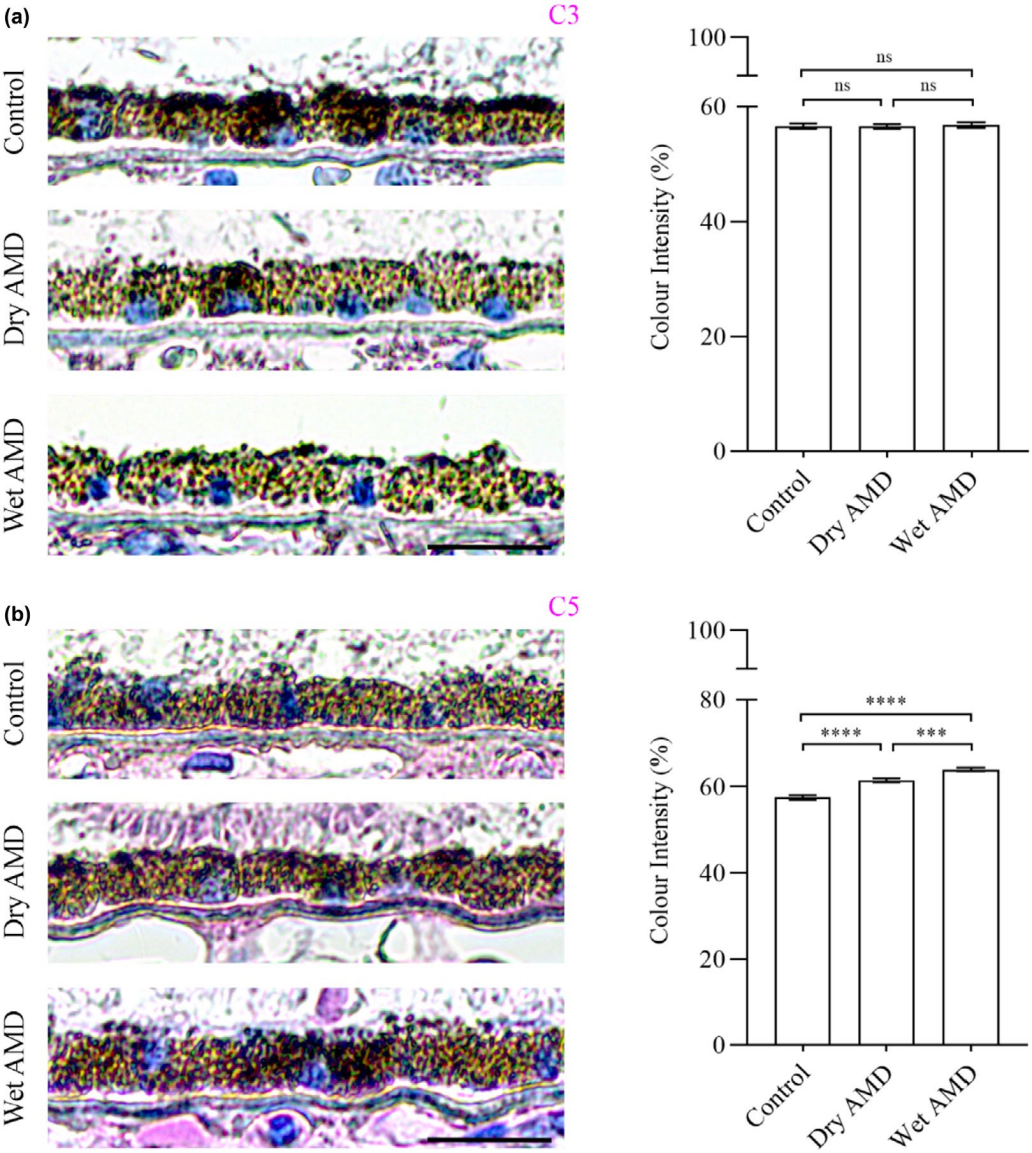
Immunohistochemical analysis of C3 and C5 in the RPE cells from control, dry AMD and wet AMD tissue samples. (a) There were no differences in the levels of C3 between control, dry and wet AMD. (b) C5 levels were significantly increased in dry (~6.9%) and wet (~11.3%) AMD compared to controls, with a significant ~4% increase in wet AMD compared to dry AMD. Scale bar = 5 μm. ****p = 0.001, ***p = 0.009, ns, not significant.

**FIGURE 13:**
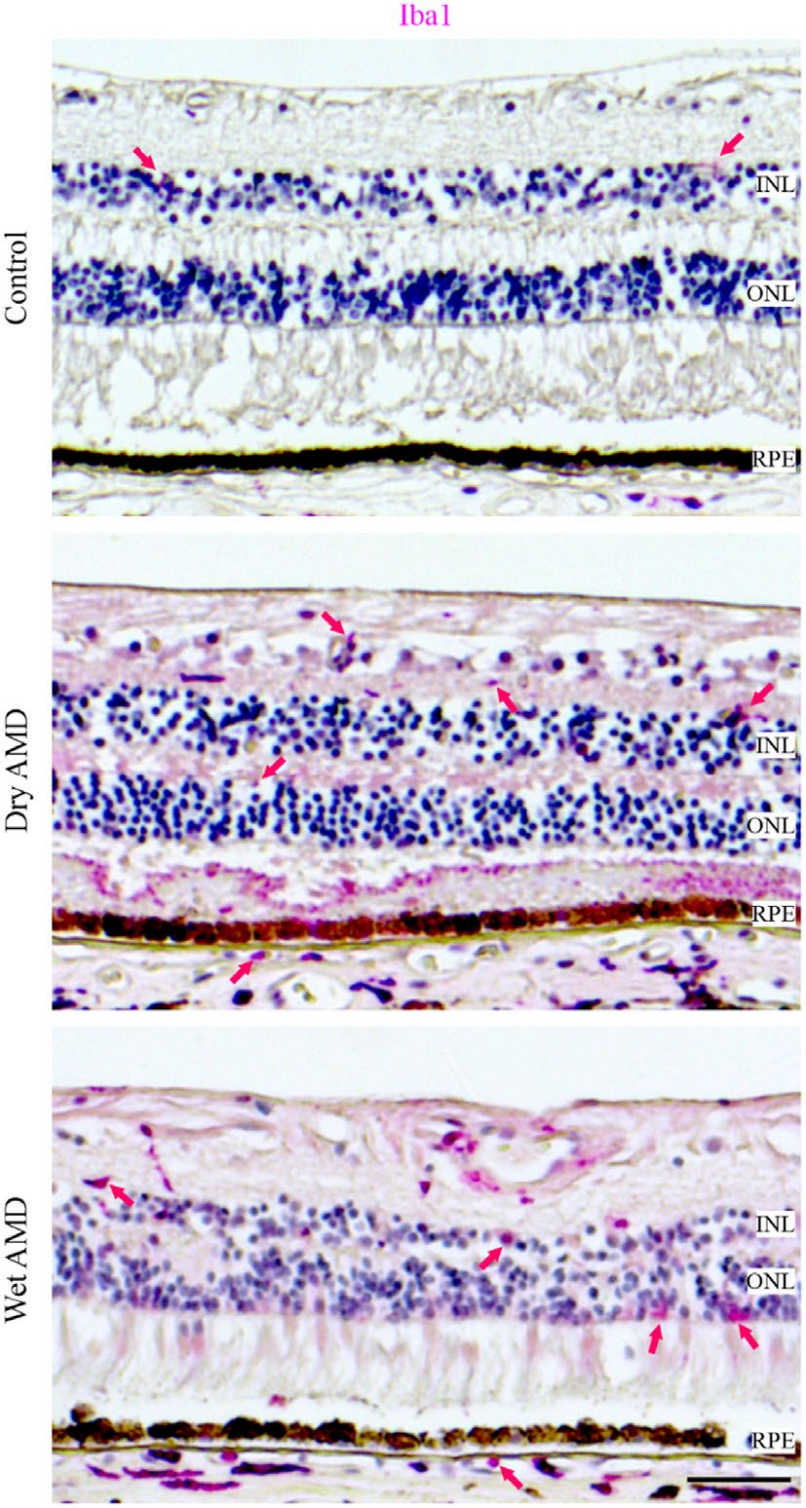
Immunohistochemical analysis of Iba1 expression was performed on retinal tissue from control, dry AMD and wet AMD samples. Increased Iba1‐positive cell staining was observed in both dry and wet AMD compared to control retina. INL, inner nuclear layer; ONL, outer nuclear layer; RPE, retinal pigment epithelial cells. Scale bar = 100 μm.

## 1 DISCUSSION

Overall, the observed increases in ubiquitin and galectin‐8 indicate the accumulation of damaged or non‐functional proteins and organelles. Elevated levels of autophagy‐related proteins, such as p62/SQSTM1 and Beclin‐1, suggest an upregulation of the degradative autophagy pathway. However, recent studies (Kaarniranta et al., 2023) show that increased oxidative stress can overwhelm and exhaust this pathway. Consistent with these findings, the build‐up of damaged cellular components in both dry and wet AMD suggests that the degradative autophagy pathway lacks sufficient clearance capacity under stress in the RPE cells.

In response, the secretory autophagy pathway may become activated to alleviate cytoplasmic burden. This is supported by increased expression of TRIM16, SEC22B, ferritin, IL‐1β, HMGB1, amyloid‐β and vitronectin. Secretory autophagy enables the translocation of intracellular components, such as amyloid‐β, to the extracellular space and their participation in the drusen biogenesis (Hyttinen et al., 2024).

Moreover, elevated levels of the tight junction protein ZO‐1, along with mesenchymal markers N‐cadherin and fibronectin, suggest that the RPE cells adopt a non‐canonical or partial EMT phenotype. This is further supported by increased expression of EMT regulators such as TGF‐β2, VEGF and SLUG. Although traditional EMT typically involves a reduction in epithelial proteins like ZO‐1, its upregulation here may reflect a compensatory response or a unique, partial EMT state in AMD.

In the complement system, elevated C5 levels—despite unaltered C3—in the RPE cells from both dry and wet AMD may reflect rapid C5 turnover or activation at a later stage. Notably, C5 can be activated independently of C3, possibly via thrombin, as shown in an AMD‐like animal model (Sridevi Gurubaran et al., 2021). Additionally, the increased number of Iba1‐positive cells was mostly confined to the inner retina, with only a few cells observed in close proximity to the RPE cells, suggesting that immune cell infiltration near the RPE is limited in our samples. This may indicate a secondary inflammatory response.

Together, these findings suggest that secretory autophagy contributes to both drusen formation and EMT during AMD progression and may potentially open new avenues for therapeutic intervention.
